# Mechanisms of Sodium Transport in Plants—Progresses and Challenges

**DOI:** 10.3390/ijms19030647

**Published:** 2018-02-25

**Authors:** Monika Keisham, Soumya Mukherjee, Satish C. Bhatla

**Affiliations:** 1 Laboratory of Plant Physiology and Biochemistry, Department of Botany, University of Delhi, Delhi 110007, India; monik.kim@gmail.com (M.K.); soumobios@gmail.com (S.M.); 2 Department of Botany, Jangipur College, University of Kalyani, West Bengal 742213, India

**Keywords:** sodium influx, ouabain-sensitive ATPase, sodium efflux, ouabain, Na^+^, K^+^-ATPase

## Abstract

Understanding the mechanisms of sodium (Na^+^) influx, effective compartmentalization, and efflux in higher plants is crucial to manipulate Na^+^ accumulation and assure the maintenance of low Na^+^ concentration in the cytosol and, hence, plant tolerance to salt stress. Na^+^ influx across the plasma membrane in the roots occur mainly via nonselective cation channels (NSCCs). Na^+^ is compartmentalized into vacuoles by Na^+^/H^+^ exchangers (NHXs). Na^+^ efflux from the plant roots is mediated by the activity of Na^+^/H^+^ antiporters catalyzed by the salt overly sensitive 1 (SOS1) protein. In animals, ouabain (OU)-sensitive Na^+^, K^+^-ATPase (a P-type ATPase) mediates sodium efflux. The evolution of P-type ATPases in higher plants does not exclude the possibility of sodium efflux mechanisms similar to the Na^+^, K^+^-ATPase-dependent mechanisms characteristic of animal cells. Using novel fluorescence imaging and spectrofluorometric methodologies, an OU-sensitive sodium efflux system has recently been reported to be physiologically active in roots. This review summarizes and analyzes the current knowledge on Na^+^ influx, compartmentalization, and efflux in higher plants in response to salt stress.

## 1. Introduction

**Figure 1:**
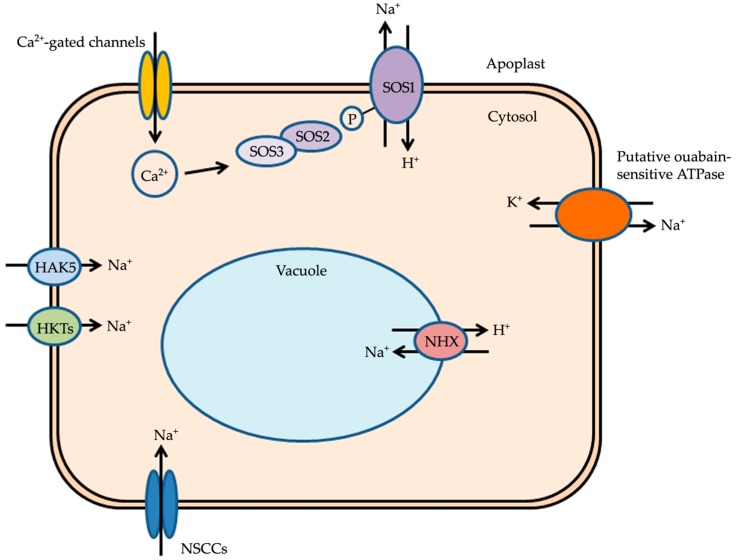
An overview of the mechanisms of Na^+^ influx, compartmentalization of Na^+^ in vacuoles, and Na^+^-sensing followed by Na^+^ efflux in response to salt stress. Na^+^ enters the cell via channels such as NSCCs and transporters such as HAK5 and HKTs. Na^+^ is sensed by an unidentified sensory mechanism, and Ca^2+^ signaling cascade is activated. The salt stress-induced increase in cytosolic concentration of Ca^2+^ is sensed by SOS3 (a myristoylated Ca^2+^ sensor protein). SOS3 interacts with SOS2 and activates its kinase activity. The SOS2–SOS3 complex becomes localized to the plasma membrane. SOS2 then phosphorylates SOS1 and activates its Na^+^/H^+^ antiporter activity facilitating Na^+^ efflux from the cell. Additionally, Na^+^ is effluxed through a novel efflux mechanism involving a putative ouabain (OU)-sensitive ATPase. Na^+^ is also compartmentalized inside vacuoles by NHX (a Na^+^/H^+^ exchanger) as a response to salt stress. Abbreviations: NSCCs, non-selective cation channels; HAK5, high-affinity potassium transporter 5; HKTs, high-affinity potassium transporters; SOS, salt overly sensitive; NHX, Na^+^/H^+^ exchanger.

Soil salinity affects agriculture globally. Salinization can occur as a result of natural causes, such as the close proximity to coastal areas, or be anthropogenic, due, for instance, to poor irrigation practices that lead to the accumulation of high concentrations of salts. Saline soils contain high concentrations of salts such as CaSO_4_ and Na_2_CO_3_, although NaCl is the dominant salt [1]. The primary effects of salinity on plants are: (1) the osmotic effect leading to a water deficit due to high concentrations of solutes present in the soil; (2) ion-specific stresses leading to K^+^ deficiency due to altered K^+^/Na^+^ ratios [2]. Alteration of the K^+^/Na^+^ ratio is due to the increase in the influx of Na^+^. Under saline conditions, Na^+^ influx is facilitated through pathways that generally function for K^+^ influx, as the ionic radii of Na^+^ and K^+^ in their hydrated forms are similar, making the discrimination between the two ions difficult. As a result of this failure in discrimination, plants growing in saline soils suffer from Na^+^ toxicity and K^+^ deficiency. The toxic levels of Na^+^ present in the cytoplasm at high concentrations must be lowered in order to maintain a low cytosolic Na^+^ concentration and a high K^+^/Na^+^ ratio by mechanisms that function to: (1) reduce Na^+^ influx into root cells; (2) compartmentalize Na^+^ into vacuoles; (3) increase Na^+^ efflux from root cells [3,4]. These processes of Na^+^ detoxification and cellular osmotic adjustment are important for plants to tolerate salt stress [5]. The present review discusses the progresses made so far in understanding the mechanisms of sodium transport, such as Na^+^ influx in the roots via non-selective cation channels (NSCCs), its effective compartmentalization in vacuoles via Na^+^/H^+^ antiporters (NHX), and Na^+^ efflux from the roots upon sensing salt stress via the salt overly sensitive (SOS) pathway (Figure 1). More importantly, this review provides a detailed account of the challenges in the quest for evidence for the presence of a putative ouabain (OU)-sensitive ATPase (similar to the Na^+^, K^+^-ATPase of animals) that facilitates Na^+^ efflux in higher plants (Figure 1).

## 2. Physiological Effects of Salt Stress

Salt stress affects various physiological and metabolic processes and may eventually impede crop production depending on the extent and severity of the stress [6]. In the early stages, a high concentration of solutes present in the soil brings about osmotic stress which reduces the capacity of root systems to absorb water and, meanwhile, accelerates the loss of water from the leaves [2]. This is accompanied by ion-specific effects that cause the accumulation of toxic concentrations of Na^+^ and Cl^−^ in the cells, which manifest in the form of chlorosis and necrosis of the leaves [7]. The accumulation of internal solutes at low and moderate salt stress conditions may assist in overcoming osmotic stress in some plants [8]. A high salt concentration brings about similar deficiency symptoms as those caused by nutrient deficiency, because of the interference of ions in membrane functions, which affect the absorption of nutrients and the solute balance inside the cell [9]. Domestic and wild germplasms vary in their salt stress tolerance level, and this gives rise to varying levels of plant growth limitation among cultivars, varieties, and species [8,10]. Salt-tolerant and -sensitive varieties differ in their ability to sequester salt in vacuoles. Salt-tolerant varieties rapidly sequester salt in vacuoles, which slowly leads to alteration in their vital cellular functions. On the other hand, salt-sensitive varieties are unable to sequester salt in vacuoles and the salt accumulates rapidly in the cytoplasm followed by a reduction of photosynthesis and assimilation [2,11]. Salt stress causes various physiological changes such as: (1) decrease in the rate of photosynthesis [2,12,13,14,15]; (2) smaller stomatal aperture and lower stomatal conductance due to disturbed water relations and sensitivity to abscisic acid (ABA) [2,12,13,15,16,17]; (3) decrease in transpiration rate [12,18,19]; (4) decrease in chlorophyll a and b, total chlorophyll, and carotenoids concentrations [14,17,20,21,22,23]; (5) decrease in chlorophyll fluorescence [15,24,25,26]; (6) changes in leaf anatomy, such as a decrease in the thickness of the epidermis and mesophyll and a decrease in intercellular spaces in the leaves [27,28], and a reduction in root length density [29,30]; (7) nutrient imbalance with a decrease in the content of phosphorus, nitrogen, Ca^2+^, Mg^2+^, and K^+^ [21,31]; (8) decrease in leaf relative water content [8,32,33,34,35]; (9) membrane instability and increase in membrane permeability [35,36,37,38].

## 3. Mechanism of Sodium Influx into the Cytosol

Plants generally maintain a high cytosolic K^+^/Na^+^ ratio and a negative electrical membrane potential difference (around −140 mV) across the plasma membrane under a normal physiological state. However, under salt stress conditions, the increase in Na^+^ concentration in the soil establishes an electrochemical gradient that favours the transport of Na^+^ passively from the soil into the cytosol [5]. Na^+^ influx into the roots occurs through different channels and transporters (Table 1). The channels involved in Na^+^ influx are the non-selective cation channels (NSCC), including the cyclic nucleotide-gated channels (CNGCs) and glutamate receptors (GLRs). The transporters involved in Na^+^ influx are high-affinity potassium transporters (HKTs and HAKs) [3,39,40,41,42]. Aquaporins have also been recently reported to be involved in Na^+^ uptake in plants [43]. From the root, Na^+^ is transported into the xylem by other channels and transporters and then delivered to the shoot [2]. Voltage-insensitive NSCCs (VI-NSCCs) are considered to be an important route for influx of Na^+^ into the roots and hence have been studied with regard to salt stress tolerance. There are two subgroups of VI-NSCCs categorized on the basis of the blocking effect of cations, such as Ca^2+^, Zn^2+^, Mg^2+^, and Ba^2+^. The cations can freely pass through the channels of one group whereas the other group is partly blocked by these cations [39]. Quinine is inhibitory to some VI-NSCCS [44] but not to others [45]. There is relevant evidence that the influx of Na^+^ into roots occurs mainly via VI-NSCCs [3,46,47,48,49]. The permeability of Na^+^ through VI-NSCCs has been demonstrated in endosperm cells of *Haemathus* or *Clivia* fruits [50], epidermal cells of *Pisum sativum* leaves [51], protoplasts of rye roots [45], suspension culture cells of barley [52], symbiosome membrane of *Lotus japonicus* [53], protoplasts of wheat root cortex [54], root cells of *Arabidopsis* [46], and protoplasts of *Arabidopsis* roots [44]. These observations are additionally validated by studies that report that Na^+^ influx into intact tissues via NSCCS is partially inhibited by Ca^2+^ and it is sensitive to its blockers such as quinine [55,56]. Additionally, two subclasses of NSCCs have been proposed to be involved in Na^+^ influx, namely, CNGCs and GLRs. *Arabidopsis* genome has 20 each of CNGCs and GLRs [57]. In *Arabidopsis* roots, AtCNGC3 has been reported to be involved in Na^+^ fluxes. Tissue expression analysis using β-gluconidase (GUS) has predominantly localized AtCNGC3 to root cortical and epidermal cells. A null mutation in AtCNGC3 has been reported to reduce the net uptake of Na^+^ during the early stages of exposure to NaCl (40–80 mM). However, longer exposure of the wild-type (WT) and mutant seedlings to NaCl (80–120 mM) leads to the accumulation of similar Na^+^ concentrations in both plants [58]. These results indicate the involvement of AtCNGC3 in the uptake of Na^+^ during the early stages of salt stress. In salt-tolerant rice varieties, OsCNGC1 is downregulated to a higher extent than in salt-sensitive varieties under salt stress conditions [59]. Several reports suggest that unidirectional Na^+^ flux or net fluxes are sensitive to cyclic nucleotides which support the involvement of CNGCs in the transportation of Na^+^ in plants [48,55]. These investigations provide subtle hints for the involvement of CNGCs in Na^+^ fluxes. GLRs are ligand-sensitive NSCCs gated by glutamate that may be involved in Na^+^ fluxes in plants [60]. Patch clamp experiments using *Arabidopsis* root protoplasts detected voltage-insensitive Na^+^ and Ca^2+^ currents activated by glutamate [61]. The other evidence for the possible involvement of GLRs in Na^+^ uptake comes from experiments using *Xenopus laevis* oocytes. The expression of *AtGLR3.7* in the oocytes promoted Na^+^ permeable conductance [62]. The ion pore domains of AtGLR1.1 and AtGLR1.4 have been demonstrated to mediate Na^+^ transport [63]. The present findings point to a possible involvement of CNGCs and GLRs in Na^+^ fluxes and uptake in plants, and their roles cannot be denied at this moment, but extensive and careful investigation is necessary to clearly show their significant involvement in primary Na^+^ fluxes.

The high-affinity K^+^ transporters (HKTs) are members of a family of transporters found in bacteria, fungi, and plants [86]. There are two classes of HKTs that differ in their specificities for Na^+^ and K^+^. Class I HKTs are Na^+^-specific transporters with the SGGG (S = serine, G = glycine) pore-forming motif present in the polypeptide and are found in both monocotyledons and dicotyledons. Class II HKTs are Na^+^–K^+^ co-transporter with the GGGG pore-forming motif present in the polypeptide and are found in monocotyledons [41,87]. AtHKT1;1 is a member of class I HKT s and is the best characterized member from this class. AtHKT1;1, isolated from *Arabidopsis thaliana,* mediates Na^+^ influx in heterologous expression systems [64]. Apparently, there is a salt stress tolerance determinant that controls Na^+^ influx into roots, thereby resulting in lower Na^+^ accumulation in *athkt1;1* mutants than in WT plants [65]. However, some investigations have reported that AtHKT1;1 is a part of the salt stress tolerance mechanism involved in Na^+^ recirculation by phloem [37] and retrieval of Na^+^ from the xylem [66], but it does not contribute to Na^+^ influx. The involvement of AtHKT1;1 in Na^+^ uptake in roots has been shown in plants exposed to the soil bacteria *Bacillus subtilis* GB03. The contribution of AtHKT1;1 to low-affinity Na^+^ uptake has recently been demonstrated in *A. thaliana*. Tetraethylammonium ((TEA^+^), an inhibitor of some K^+^ transporters and most K^+^ channels) and ammonium ions ((NH_4_^+^), an inhibitor of HAK5) do not bring about any significant influence on ^22^Na^+^ influx in WT plants but reduce the influx in *athkt1;1* plants. The net Na^+^ uptake rate is higher in both WT and *athkt1;1* plants exposed to 25 mM NaCl and 0.01 mM K^+^ than in those exposed to 2.5 mM K^+^ alone [56]. The rice OsHKT2;1 is a class II HKT with the SGGG motif instead of the GGGG motif of a typical class-II HKT transporters. Horie et al. (2007) demonstrated that OsHKT2;1, unlike traditional class-II HKTs, mediates influx of Na^+^ into the root cells [40]. Plants lacking *OsHKT2;1* gene, when exposed to 0.5 mM Na^+^ in the absence of K^+^, have lower Na^+^ accumulation and reduced growth [40]. OsHKT2;2/1 is a novel HKT isoform isolated from roots. It is an intermediate between OsHKT2;1 and OsHKT2;2 that was earlier believed to confer salt tolerance to the rice cultivar Nona Bokra by enabling K^+^ uptake in roots under salinity stress [67]. OsHKT2;2/1 has now been shown to mediate Na^+^ influx into the roots of plants exposed to salinity stress [88]. The K^+^ uptake permeases KUP/HAK/KT is a family of K^+^ transporters. *PhaHAK5* is expressed only in reed plants that are sensitive to salt. Yeast strains expressing *PhaHAK5* show Na^+^ permeability and poor K^+^ uptake ability under salt stress [68]. AtHAK5 might be involved in low-affinity Na^+^ uptake regulated by K^+^ concentrations in *A. thaliana*. Roots of *athkt1;1* plants exposed to 25 mM NaCl plus 2.5 mM K^+^ exhibit higher transcript levels of *AtHAK5* than WT roots. *AtHAK5* transcript levels also increase continually during 48 h in *athkt1;1* roots exposed to 25 mM NaCl plus 0.01 mM K^+^ [56]. The probable role of HAK5 in low-affinity Na^+^ uptake has so far been only reported from reed plants and *Arabidopsis.* HKTs not only mediate Na^+^ influx but also have different functions in Na^+^ transport processes in different species. Further careful investigations on these aspects will be helpful in dissecting the clear role of these K^+^ transporters in Na^+^ transport as well as their function in enhancing salt stress tolerance.

## 4. Mechanism of Sodium Influx into the Vacuoles

Plants compartmentalize Na^+^ into the vacuoles of most tissues so as to reduce the toxic concentration of Na^+^ in the cytosol. Vacuolar Na^+^/H^+^ exchangers (NHXs) are considered to be an important regulator of this process. *Arabidopsis* NHX1 (AtNHX1), the first NHX identified in plants, is localized to tonoplast [70], and constitutive overexpression of *AtNHX1* in *Arabidopsis* improves salt stress tolerance [89]. Morever, overexpression of *NHX1* in various transgenic plants, such as *Brassica* [71], cotton [72], maize [73], rice [74], tobacco [75], tomato [76], and wheat [77] exposed to NaCl stress ranging from 100 mM to 200 mM NaCl concentrations enhances their salt stress tolerance levels. In *Arabidopsis*, NHX1 and NHX2 are the most abundant NHXs and have recently been reported to be involved not only in Na^+^ compartmentalization but also in other processes, including uptake of K^+^ into the vacuoles and regulation of vacuole pH [78,90]. Induction of NHX1 and NHX2 in response to salt stress is ABA-dependent [78,91]. NHX2 and NHX5 have been reported to be the determinants of salt stress tolerance, but their direct role in Na^+^ sequestration is not clear till date [78,92]. It is widely accepted that, during salt stress, the activity of NHXs increases, which promotes salt stress tolerance in many plants [93]. Slow vacuolar (SV) channel is encoded by the *TPC1* gene in *Arabidopsis* [94]. SV channels might be involved in Na^+^ transport across the tonoplast as they can take up Na^+^ in vacuoles, but they do not mediate Na^+^ release even in the presence of a 150-fold Na^+^ gradient in the direction from the lumen to cytosol [95].

## 5. Long-Distance Transport of Sodium

Once Na^+^ from the soil traverses the epidermis, it is radially transported to the xylem via the apoplastic and/or symplastic pathways [57,60]. A part of the Na^+^ moving through the apoplastic pathway is bound to the root apoplast, and the rest generally reaches the endodermis beyond which transport across the plasma membrane is necessary for further radial transport [60]. Apoplastic barriers such as Casparian strips and suberin lamellae in the endodermis and exodermis facilitate a highly selective ion movement into and out of the stele. However, in some instances, Na^+^ might cross the endodermis through the apoplastic pathway via a bypass flow. The bypass flow is a bypassing of symplast in regions where aploplastic barriers are underdeveloped or lacking, such as in the young roots and root tips or at the site of origin of lateral roots. The transport of Na^+^ to the shoot by bypass flow is significant in rice but not in *Arabidopsis* [55,96,97,98]. Silicon supplementation can reduce the apoplastic bypass flow of Na^+^ [99,100]. Another physical barrier to Na^+^ transport via the apoplastic pathway reported in *Brassica* plants is represented by phi cells with a thick cell wall which prevent Na^+^ movement into the xylem [101]. In both halophytes and glycophytes, enzymes in the apoplast are more salt-tolerant than those in the cytoplasm, which helps apoplast withstand high Na^+^ concentrations [102]. The proteome of the rice root apoplast is altered by salt stress, which can be a response to the osmotic effect or to the Na^+^-specific effect of salt stress [103,104,105]. However, further investigations are necessary to study whether the changes in protein expression levels have an effect on Na^+^ transport directly or whether they are involved in Na^+^-sensing. Na^+^ that enters the root cytoplasm through different channels and transporters, as discussed earlier, moves rapidly to the xylem of the root stele through the symplastic pathway connected by plasmodesmata. It is not known whether Na^+^ transport via the symplast is sensed or regulated [60]. Although no transporter involved in the radial transport of Na^+^ has been characterized, a member of the cation/H^+^ exchanger (CHX) family in *Arabidopsis*, namely, CHX21, is a possible candidate. CHX21 is expressed in root endodermal cells, and its mutants under salt stress accumulate less Na^+^ in their xylems and leaf sap indicating that CHX21 may be involved in Na^+^ transport across the endodermis into the stele [106]. An *Arabidopsis* mutant called *sas1* overaccumulates Na^+^ in its shoot and has impaired root radial transport of Na^+^ [107]. Once Na^+^ reaches the stele, it is loaded into the xylem and enters the transpiration stream for long-distance transport from the root to the shoot. Transporters present in the plasma membrane are proposed to be involved in xylem loading of Na^+^ [108]. Like the apoplastic barrier, this is an important point where plants can control Na^+^ translocation from the roots to the shoot. At moderately saline conditions, SOS1 most likely mediates xylem loading of Na^+^, as Na^+^ is accumulated to a lesser extent in the *sos1* mutants [109]. The involvement of SOS1 in xylem loading of Na^+^ is substantiated by experiments performed in *Plantago maritima* and *Hordeum vulgare* that showed that the acidification of xylem leads to a rise in Na^+^ concentration in the xylem sap [79]. At high saline conditions, xylem loading of Na^+^ is most likely to be a passive process, as a high cytosolic Na^+^ concentration in the xylem parenchyma cells and a comparatively depolarized plasma membrane would favour the movement of Na^+^ into the xylem [110]. Plants can retrieve Na^+^ from the xylem into root cells so as to prevent high concentrations of Na^+^ in the above-ground tissues [108]. This retrieval has been observed to occur in the basal regions of the roots and shoots of plants such as maize, bean, and soybean [3,79]. AtHKT1;1 plays a key role in xylem unloading. In *Arabidopsis*, mutation in *HKT1* renders the mutants hypersensitive to salt stress and leads to higher accumulation of Na^+^ in the leaves [66,69,111]. Knockout lines exhibit higher levels of Na^+^ but low levels of K^+^ in the shoots. These results show that AtHKT1 is involved in Na^+^ retrieval from the xylem, while directly stimulating the loading of K^+^. This is one of the mechanisms to maintain a higher K^+^/Na^+^ ratio in the shoots during salt stress in plants [112]. Similar mechanisms of Na^+^ retrieval are also reported from wheat and rice. In wheat, Na^+^ exclusion (Nax) loci Nax1 and Nax2 that probably encode HKTs participate in the retrieval of Na^+^ [113]. In rice, OsHKT1;5 retrieves Na^+^ from the xylem [114].

## 6. Sodium-Sensing and Efflux from the Root

The two primary effects of salt stress are hyperosmotic and ion specific (Na^+^) stresses. In order to respond and overcome salt stress, plants have an innate ability to sense these immediate effects of salt stress. However, the molecular identities of the hyperosmotic sensors and Na^+^ sensors are still unknown. *Arabidopsis* histidine kinase1 (HK1) has been shown to complement the loss of the yeast osmosensor synthetic lethal of N-end rule (SLN1), and overexpression lines exhibit drought stress-associated phenotypes, while loss-of-function lines exhibit osmotic stress-associated phenotypes [115,116]. Transgenic *Arabidopsis* plants overexpressing hk1 have a drought-tolerant phenotype with healthy rosette leaves and inflorescences [115]. The loss-of-function lines grown on different osmolyte-supplemented media exhibited a lower percentage of germination and root elongation than their wild-type homologs [116]. Hyperosmotic stress induces various physiological responses in plants. However, recent investigations on *hk1* mutants have shown that, although some of these physiological responses are altered, many of them remain unaffected, indicating that proteins other than HK1 are likely to be involved in the perception of osmotic stress [117]. Plants show a rapid increase in cytosolic Ca^2+^ levels within the first few seconds of exposure to osmolytes such as mannitol or NaCl. Ca^2+^ release is primarily from the apoplastic space through the activation of phospholipase C (PLC) and subsequent release from intracellular Ca^2+^ stores [118]. This rapid Ca^2+^ response in plants emerges from the roots and takes place in various cell types [119,120]. This finding has led to the hypothesis that hyperosmotic stress is likely to be sensed by a mechanically gated Ca^2+^ channel [121]. The downstream targetsof this Ca^2+^ channel, namely, kinases like calcineurin B-like proteins (CBLs), calcium-dependent protein kinases (CDPKs), and CBL-interacting protein kinases (CIPKs), may become activated. CIPK initiates a cascade of phosphorylation events, and the hyperosmotic signal is further transduced to regulate other downstream components for salt stress tolerance [122,123]. Many CBLs are reported to interact with CIPK24 in response to salt stress. CBL1 interaction with CIPK24 mediates the regulation of Na^+^ [124]. Morever, the NaCl-induced cytosolic increase in Ca^2+^ is sensed by SOS3 (a myristoylated Ca^2+^ sensor protein with three elongation factor (EF) hands for Ca^2+^ binding) [125,126,127,128]. Treatment of one-week-old *Arabidopsis* seedlings with 2-hydroxymyristic acid (HMA, a myristoylation inhibitor) leads to root tip swelling, a phenotype shown by *sos3* mutant plants. Mutations in the *SOS3* gene that reduce calcium binding or disrupt the myristoylation of the protein make *Arabidopsis* plants hypersensitive to NaCl stress, thereby suggesting that both calcium binding and myristoylation are necessary for SOS3 role in NaCl stress tolerance [128]. SOS3 transduces the NaCl stress-induced calcium signal by interacting with SOS2, a serine/threonine kinase belonging to the SnRK3 (a sucrose non-fermenting-1 (SNF1)-related protein kinases) family [129,130]. In *Arabidopsis*, *SOS2* is expressed in both root and shoot tissues, while its expression in roots is upregulated by NaCl treatment [129]. SOS2 has an N-terminal catalytic domain and a C-terminal regulatory domain containing a conserved 21-amino acid FISL motif (named after the prominent conserved amino acid residues Phe-Ile-Ser-Leu). The C-terminal regulatory domain is also an auto-inhibitory domain as its FISL motif inhibits the kinase activity of SOS2. SOS2 kinase activity becomes active after it binds to SOS3 via the FISL motif in a Ca^2+^-independent manner [131], and this interaction occurs in the root [132]. In shoots, the SOS3 homolog SOS3-like calcium-binding protein 8 (SCABP8/CBL10) has been shown to interact with SOS2 in response to salt stress [132]. SOS2–SOS3 complex is then recruited to the plasma membrane where it phosphorylates SOS1 [133]. *GIGANTEA* (*GI*) is a flowering time gene which interacts with SOS2 and inhibits SOS2 interaction with SOS3. This inhibition by GI does not allow SOS1 to be phosphorylated in the absence of salt stress. However, in salt stress conditions, GI is degraded and SOS2 is freed making it available for interaction with SOS3, so that SOS1 can be activated to enhance salt stress tolerance [134]. SOS1 is a Na^+^/H^+^ antiporter first identified in *Arabidopsis* by mapping of a salt hypersensitive phenotype using lack of root bending on NaCl-containing media as a screen. In *Arabidopsis*, *SOS1* shows expression in the epidermal cells at the root cap and in the cells around the xylem [135]. NaCl stress leads to the upregulation of *SOS1* expression in both *Arabidopsis* root and shoot tissues [109]. The concentration of Na^+^ is threefold higher in both roots and shoots of *atsos1* mutants [66]. In *Arabidopsis*, overexpression of *SOS1* leads to reduced accumulation of Na^+^ in the xylem and shoots [80]. These investigations using *Arabidopsis sos1* mutants have not directly measured Na^+^ efflux, however the expression of *SOS1* in root cells, the subsequent upregulation of its expression by NaCl stress, and the increase in roots as well as in shoots of Na^+^ concentration in the mutants indicate that SOS1 plays an important role in Na^+^ efflux in the roots of plants.

## 7. Quest for a Na^+^, K^+^-ATPase in Higher Plants

Na^+^ efflux systems in plants and animals are exclusive in nature in terms of their structure and mode of action. The current understanding of the mechanisms of Na^+^ exchange across plant cell membranes has been supported by various reports of H^+^-ATPase-energized ion transport mechanisms and Na^+^/H^+^ antiporters [136]. H^+^-ATPase is the major P-type ATPase present in plant membranes which supports the bioenergetic system of ATP hydrolysis coupled to H^+^ transport. Na^+^, K^+^-ATPase was the first kind of P-type ATPase reported in animal system [137]. However, investigations in plants have failed to report similar observations. Recent investigations for the presence of P-type ATPase genes in various plant groups have demonstrated the existence of Na^+^ pumps in lower plants [138]. Higher plant groups also possess ouabain (OU)-sensitive Na^+^ transport mechanisms [139]. The Na^+^- and H^+^-ATPases mutually exclude each other in higher plants [90]. Interestingly, however, algae and bryophytes (lower plant groups) have been reported to possess both Na^+^- and H^+^-energized ATPases. There are reports suggesting the omission of Na^+^ pumps during the evolution of higher plants [138]. Ancestral members of higher plants might have possessed functional Na^+^-ATPase coinciding with their aquatic habitat. The quest for Na^+^-, K^+^-ATPases in higher plants has provided evidence for ouabain-sensitive ion transport mechanisms. The evolution of P-type ATPases in higher plants does not exclude the possibility of the presence of Na^+^ transport mechanisms similar to Na^+^, K^+^-ATPase in animals. The evidence for putative oubain-sensitive ATPases implies the presence of ouabain-senstive Na^+^ regulators associated with plant membranes. Chlorophyte (green algae) members have been reported to possess gene sequence similar to Na^+^, K^+^-ATPase [140,141,142]. Biochemical evidence suggests the presence of vanadate and ouabain sensitivity among these ATPases in chlorophytes [140]. The present review also focuses on understanding the evolutionary fate of Na^+^-, K^+^-ATPases in plants.

## 8. Structure of the Ouabain-Sensitive Na^+^, K^+^-ATPase in Animal Cells

**Figure 2:**
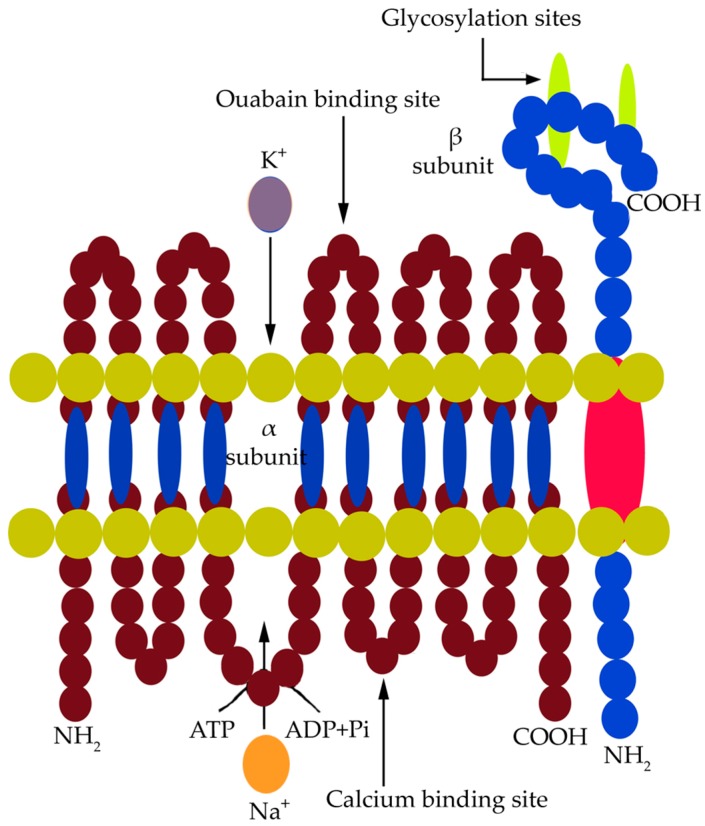
Probable structure of the α and β subunits of Na^+^, K^+^-ATPase in animal cells [143].

Na^+^, K^+^-ATPase in animals is sensitive to the glycoside ouabain. This electrogenic P-type membrane pump carries out a coupled transport of three Na^+^ ions outside the cell and two K^+^ ions inside the cell. It is a transmembrane heterodimeric protein comprised of α and β subunits, forming intracellular and extracellular domains and five pairs of transmembrane helices [95]. The α and β subunits have four isoforms each, expressed in a tissue- and cell-specific manner, and they vary in their sensitivity towards Na^+^ and the steroid glycoside ouabain (inhibitor of Na^+^, K^+^-ATPase in animals). The pump carries out a coupled ion transport accomplished by the hydrolysis of ATP and a subsequent phosphorylation event. It requires Mg^2+^-ATP as its major substrate and has affinity towards specific ligands [143]. The α subunit is 110 kDa in size and comprises about 1000 amino acid residues. The four isomers of the α subunit, namely, α_1_, α_2_, α_3,_ and α_4_ differ in their functional properties. The α_1_ subunit is a housekeeping isoform expressed in most animal tissues [143]. A saline environment triggers the quantitative expression of specific isoforms depending upon their Na^+^-binding affinity. The α subunit consists of five pairs of transmembrane helices forming the cation transport path and of three cytoplasmic domains which contain the binding sites for Mg^2+^-ATP and Na^+^ (Figure 2).

These cytoplasmic domains are specified as nucleotide-binding sites, phosphorylation sites, and an actuator site. The transmembrane helices move or rotate to transport ions across the membrane. The α subunit is an active component of the pump carrying out phosphorylation and ion transport [143]. The β subunit is 55 kDa in size and is composed of 370 amino acid residues, of which 30 amino acids form a cytosolic loop, and the rest are folded to form the extracellular domain. The β subunit has three consecutive glycosylation sites on its extracellular folds. This subunit does not contain any active site for the substrate. It, however, interacts with the α subunit to restore the native structure of the enzyme. There are three S–S bonds in the extracellular folds of the subunit necessary to provide the K^+^ occlusion state of the enzyme. The pump operates through a cycle of phosphorylation–dephosphorylation events accompanied by Na^+^ and K^+^ binding and hydrolysis of ATP. Na^+^ binding at the cytosolic loop of the α subunit initiates Na^+^-dependent phosphorylation of the pump, accomplished by the hydrolysis of ATP bound to it [143]. This results in the formation of an acyl-phosphate complex between the inorganic phosphate (Pi) and a specific aspartate (Asp) residue. This phosphorylated state of the pump, commonly stated as (E_1_P), now excludes three Na^+^ ions from the cell. Two K^+^ ions then bind to the extracellular fold of the enzyme, and the enzyme attains the (E_2_P) state [143]. This is followed by occlusion of K^+^ into the cell and subsequent dephosphorylation of the pump. Ouabain is a specific glycoside which can bind to the phosphorylated α subunit of the enzyme in the (E_2_P) state after it has excluded three Na^+^ ions [143].

## 9. Inhibition of the Ouabain-Sensitive ATPase by Calcium

Ca^2+^ is a regulator of Na^+^ transportation by Na^+^, K^+^-ATPase. Ca^2+^ concentration at the physiological range of 0.08–5 µM inhibits the activity of Na^+^, K^+^-ATPase [144]. The excitation of cells leads to depolarisation which increases the cytosolic Ca^2+^ concentration beyond the threshold level and subsequently causes inhibition of certain isoforms of the Na^+^, K^+^-ATPase in a concentration- and affinity-dependent manner. However, the Na^+^, K^+^-ATPase activity can be inhibited altogether irrespective of the isomer by high concentrations of Ca^2+^. The α subunit of the enzyme is associated with Ca^2+^ inhibition, with the α_2_ isomer having greater affinity for Ca^2+^ at physiological levels of 0.08–5 µM and even at higher concentration of 10 mM [144]. Ca^2+^ at the concentration of 10^−5^ M competes with Mg^2+^ for ATP and lowers the concentration of Mg^2+^-ATP, which is a rate-limiting substrate for the enzyme. Two Ca^2+^-binding proteins, namely, calmodulin and calnaktin are known to mediate the process of Ca^2+^ inhibition of this enzyme [145,146]. Calmodulin and calnaktin lower the threshold level of Ca^2+^-mediated inhibition from 100 µM to 2–5 µM of Ca^2+^. These proteins are unable to inhibit the pump in the absence of Ca^2+^. Ca^2+^-dependent inhibition of the pump is initiated by calmodulin and calnaktin, and they interact with at least one or more proteins present in the plasma membrane or cytosol to bring about the inhibition of the pump. The process of Ca^2+^ inhibition associated with the α subunit of the enzyme is noncompetitive and it does not compete with Na^+^ for its binding site.

## 10. Ouabain-Sensitive ATPases in Plants: A Physiological Enigma

Ouabain (OU) is a well-known cardiac glycoside used as a specific inhibitor of Na^+^, K^+^-ATPase activity in animals [147]. Speculating that plants possess OU-sensitive ATPases, plant scientists have reported the presence of putative OU-sensitive sodium efflux mechanisms similar to those mediated by Na^+^, K^+^-ATPase in animals [139]. However, further investigations did not support these findings because of the lack of suitable methodologies available in late nineties. Genomic sequence data confirmed the absence of Na^+^, K^+^-ATPase in plants. This, however, did not rule out the possibility of the existence of ouabain-sensitive ATPases in plants. Several investigations have reported that ouabain has physiological effects in various processes, such as in Na^+^ fluxes in the excised roots of carrots [82], stomatal regulation in the leaf epidermis of tobacco [148], pulvinar expansion in *Mimosa pudica* [149], flowering in *Lemna gibba* [150], and transpiration rate of *Secale cereal* [151]. *Hordeum* sp. and *Halocnemum* sp. possess Na^+^, K^+^ and Mg^2+^-activated ATPases sensitive to ouabain treatment [152]. Sugarbeet roots have been reported to possess ouabain-sensitivity of ATPases [153]. Application of ouabain to excised roots of maize and barley leads to the inhibition of Na^+^ efflux from the cell, resulting in an increase of intracellular Na^+^ concentration [83,84]. Similar observations have also been reported from investigations on excised carrot roots where ouabain influenced both Na^+^ and K^+^ fluxes [82]. Ouabain-sensitive ATPases have also been reported to be present in some glycophytes and halophytes [154]. The dose-dependent response of ouabain in plant processes exhibits similarities to those in animal cells [148,155]. Lindberg (1982) reported two forms of ouabain-sensitive ATPases operative in plants, and their optimal activity has been observed in the pH range of 5.5–6.0 and at pH 8.0 [153]. The structural integrity of the ouabain binding sites of Na^+^, K^+^-ATPase provide the rationale base for investigating the evolutionary fate of Na^+^-ATPases in plants.

## 11. Salt Stress-Induced Modulation of Ouabain-Sensitive ATPase Activity: Evidence for Novel Sodium Efflux Mechanisms in Plants

**Figure 3:**
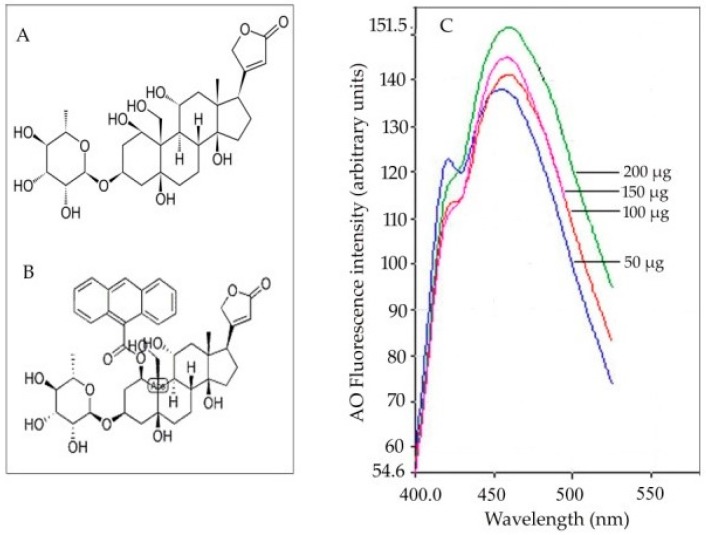
(**A**) Chemical structure of ouabain and (**B**) its fluorescent derivative 9-anthroylouabain. (**C**) Dose-dependency (protein concentration ranging from 50 to 200 µg) of fluorescence intensity obtained from the fluorescence emission spectra due to OU-sensitive ATPase binding to 9-anthroylouabain (ex. 365 nm, em. 460 nm) [85].

Recent investigations performed on sunflower seedlings have shown ouabain-sensitive ATPase activity to be correlated with the accumulation of intracellular Na^+^ ions [85]. The perception that higher plants do possess some kind of ouabain-sensitive sodium efflux systems has been substantiated by investigations on salt-stressed (120 mM NaCl) sunflower seedling roots. A novel fluorescence spectrofluorometric method has been adopted to localize and monitor the impact of salt stress on ouabain-sensitive ATPase activity in roots during the early phase of seedling growth in sunflower [85]. The methodology involves the use of 9-anthroylouabain (a fluorescent probe known to localize ouabain-sensitive ATPase activity in animal cells) for the first time in plant systems (Figure 3).

**Figure 4:**
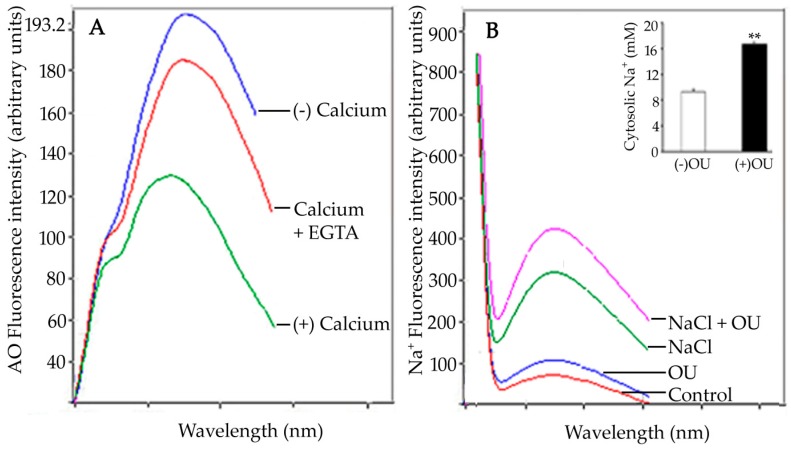

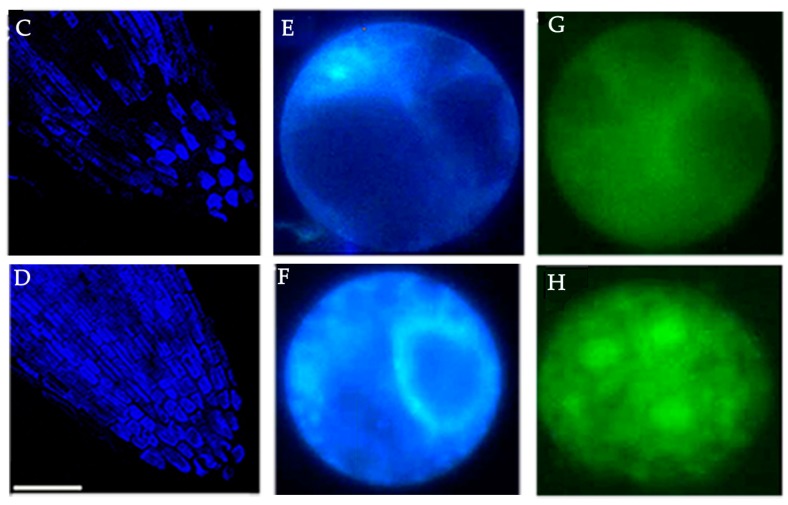
(**A**) Calcium-mediated inhibition of OU-sensitive ATPase activity and its subsequent recovery by EGTA. (**B**) Effect of ouabain (OU) on (Na^+^)_cyt_ in salt-stressed sunflower seedling roots. (**C**) Confocal laser scanning microscopic (CLSM) localization of salt stress-induced activity of OU-sensitive ATPase in two-day-old sunflower seedling root tips and (**D**) enhanced fluorescence obtained after simultaneous treatment with exogenous Ca^2+^ (10 mM) and EGTA (5 mM). Scale bar = 75 µM. (**E**) Subcellular localization of OU-sensitive ATPase from salt stress-induced seedling root protoplasts shows the distribution of the enzyme in the cytoplasmic vesicles and plasma membrane. (**F**) Enhanced enzyme activity induced by exogenous Ca^2+^ (10 mM) and EGTA (5 mM) treatment is evident in the nuclear membrane. (**G**) Effect of ouabain on Na^+^ accumulation in root protoplasts obtained from salt-stressed sunflower seedlings grown in the absence and (**H**) presence of ouabain [85].

The observed fluorescence intensity at 460 nm is a function of putative OU-sensitive ATPase activity. Enhanced fluorescence results from the binding of the enzyme to 9-anthroylouabain in a dose-dependent manner. This OU-sensitive sodium efflux system is induced in seedling roots salt stress. Confocal laser scanning microscopic (CLSM) imaging of salt-stressed seedling root tips showed significantly enhanced fluorescence due to OU-sensitive ATPase activity (as compared to control). The activity was prevalent in cells of columella, epidermis, sub-epidermis, and in meristematic cells [85]. The report suggested an inverse relation between cytosolic Na^+^ concentration and OU-sensitive ATPase activity (Figure 4).

In animal cells, the activity of ouabain-sensitive Na^+^, K^+^-ATPase is reported to be modulated by Ca^2+^. The intensity of the inhibition depends on the differential Ca^2+^ affinity for specific OU-sensitive isoforms. This differential sensitivity to Ca^2+^ is specific to tissues and environments [156]. Similar to these reports, Ca^2+^-mediated inhibition of OU-sensitive ATPase activity has also been observed in sunflower seedling roots. Root protoplasts have been used to investigate the effect of salt stress on the distribution of OU-sensitive ATPase activity. The malleability of the efflux system has been tested by ouabain treatment which leads to increased accumulation of cytosolic sodium ions (1.8 fold) in the roots of salt-stressed seedlings as a result of the inhibition of OU-sensitive ATPase activity by ouabain. Putative ouabain-sensitive ATPase activity is localized to the nuclear membrane and plasma membrane of protoplasts exposed to salt stress [85]. This localization has been attributed to the maintenance of the Na^+^–K^+^ gradient across the cytosol and nucleus [156]. Ouabain-sensitive Na^+^, K^+^-ATPase localization in animal systems has revealed a general distribution of the enzyme as evident from a diffuse blue fluorescence in the cells [157]. Ouabain-sensitive ATPase activity in *Halocnemum* sp. has been reported to increase by 238% as a result of Na^+^induction [152]. To summarize, ouabain sensitivity in various plant systems reveal its correlation with the regulation of Na^+^ transport mechanisms. Interestingly, the glycoside exhibits an inhibitory role similar to that observed for Na^+^, K^+^-ATPase in animals.

## 12. Recent Advancements in Understanding the Regulation of Sodium Transport Mechanisms in Plants: An Update

Recent investigations on the regulatory mechanisms of Na^+^ transport in plants have provided sufficient insights for salinity tolerance among various plant systems. The synergistic effects of SOS1, HKT1;5 and NHX1 have been proposed to regulate Na^+^ homoeostasis in the halophyte member *Puccinellia tenuiflora* [158]. NaCl stress-induced vacuolar compartmentalization of Na^+^ and its xylem-loading have been attributed to be regulated by thr differential expression of NHX1 and HKT1;5. Evidence, therefore, suggests the temporal and spatial exclusion of various sodium exchange mechanisms operative in this halophyte. NaCl stress-induced expression of *SOS1* and *NHX1* in the roots has also been reported to be more efficient in Na^+^ and Cl^−^ exclusion in the intertidal population of *Suaeda salsa* [81]. The genetic or environmental variation of salt tolerance among halophyte populations pertains to the differential expression of Na^+^ efflux channels. Detailed structural analysis of HKT1;5 has been performed in *Triticum* sp. [159]. Variations in its amino acid sequences result in altered Na^+^ affinity and subsequent change in the salt tolerance among two species of *Triticum*. Comparative analysis of the antioxidative mechanisms in *Cynodon dactylon* (salt-tolerant grass) and *Oryza sativa* (salt-sensitive plant) has been substantiated by the high expression levels of SOS 1 and NHX1 transporters in *Cynodon* [35]. Such investigations suggest a source of potential gene pool for high-affinity Na^+^ efflux transporters among salt-tolerant Poaceae members. A reverse genetic approach with gene silencing in near-isogenic lines has suggested a significant role of *HKT1;2* gene in the salt stress tolerance of tomato plants [160]. Interestingly, the success of the transgenic approach and salt-resistance breeding has been evaluated for the cation/proton antiporter 1 (CPA1) family candidate gene in dicotyledons and monocotyledons. CPA1 genes encode cellular Na^+^/H^+^ exchanger proteins. Among the dicotyledon members, *Arabidopsis*, however, exhibits a lesser magnitude of CPA1-induced changes due to gene transformation [161]. The possible effects of a Na^+^ efflux mechanism in plants similar to that of Na^+^, K^+^-ATPases in animals have been also been recently discussed [162]. The effectiveness of Na^+^, K^+^-ATPases in animals outstands the secondary Na^+^ antiport system in plants. The proton-mediated Na^+^ efflux system involves a considerable amount of proton drainage across plant cell membranes. A possibile proton leakage does not interfere with the functionality of Na^+^, K^+^-ATPases in animals. Considering this fact, Pedersen and Palmgreen suggested the heterologous expression of Na^+^, K^+^-ATPases from algae into higher plant cell membranes [162]. The major challenges in obtaining plant-based heterologous expression of Na^+^, K^+^ ATPases lies in the functionality of the β subunit. Na^+^, K^+^-ATPases in animals possess highly glycosylated β subunit functioning as the major catalytic domain [162]. Salt tolerance in barley has been attributed to the regulation of Na^+^ loading into xylem elements of the roots [163]. This is controlled by a cross-talk among reactive oxygen species (ROS), nicotinamide adenine dinucleotide phosphate-oxidase (NADPH oxidase), Ca^2+^, and K^+^. The modulation of *At*CCC transporter activity has been reported to have a major role in xylem loading of Na^+^ and K^+^. It is thus suggestive that salt stress elevates ROS levels in the xylem cells which, in turn, inhibits further Na^+^ entry. ROS-induced activation of K^+^ channels (SKOR) and Ca^+^ influx is, however, necessary for osmotic adjustment of the cells. The functional characterization of OsHKT1;4 in rice has revealed its preferential selectivity of Na^+^ over other cations [88]. The transporter has been reported to regulate Na^+^ efflux from stem and leaf blades of salt stress-affected rice plants. Under salt stress, transgenic rice and tobacco plants overexpressing DNA helicase *PDH45* accumulated less Na^+^ in the root and shoot as compared to the wild-type plants [164]. DNA helicase expression has been suggested to increase the hydrophobic apoplastic barrier which limits Na^+^ transport into aerial organs. Na^+^ loading and unloading from the xylem is highly regulated by the coordinated activity of HKT1;4/HKT1;5 and SOS1 [165]. The Nax1and Nax2loci in wheat have been associated with such kind of salinity tolerance. Tissue-specific differential Na^+^ accumulation is also evident as a potential aspect of salt tolerance in wheat varieties [166]. The negative regulation of salt tolerance by the *CNGC10* gene has been investigated in *Arabidopsis* [167]. There are persistent differences among Na^+^ transport mechanisms of halophytes and sensitive glycophytes [168]. The cation selectivity of ion channels and ion transporters depends on their pore diameter and amino acid composition (14). The rates of Na^+^ and K^+^ fluxes are a function of the type of active transporters and channels prevalent in the membrane of a tissue. Genetic manipulations have been possible by RNA silencing of halophytes to decipher the role of selective Na^+^/K^+^ ion transport mechanisms [168]. Protein engineering (amino acid substitution) has been performed for candidate ion transporters involved in salinity tolerance [168]. Recent investigations provide insight on the role of silicon-induced alleviation of salt stress in plants [169]. Silicon has been reported to decrease the uptake of apoplastic Na^+^ into cells, thus increasing the binding of Na^+^ to the cell walls [99]. However, silicon does not seem to alter the rate of Na^+^ efflux through SOS1 activity [169]. To summarize, recent investigations provide clues for the regulatory effect of Na^+^ transport mechanisms in various plant systems subjected to salt stress. Signaling cascades associated with Na^+^ transporters are crucial as endogenous factors regulating sodium exchange. The identification and characterization of potent salinity tolerance genes shall provide new insights to create effective transgenic crops.

## 13. Future perspectives

The molecular identities of NSCCs through which Na^+^ is influxed into the roots of plants are unknown. The role of HAK in Na^+^ influx has so far been reported only from reed plants and *Arabidopsis*. HKTs not only participate in Na^+^ influx in some plants but also have different functions in Na^+^ transport processes in different species. Future investigations on these aspects will be helpful in clearly understanding their roles in Na^+^ influx as well in salt stress tolerance. Several investigations to date substantiate the fact that ouabain affects ion transport in plant cells by modulating OU-sensitive ATPase activity through its binding with ouabain receptors. This highlights the evolutionary significance of ouabain-sensitive ATPases in plants, which is associated with sodium exchange activity. Evidence thus strengthens the hypothesis that plants seem to possess ouabain-sensitive ATPases with partial functional homology to the Na^+^, K^+^-ATPase of animals. Furthermore, OU-sensitive ATPase activity is expected to be modulated by various other biomolecules like calmodulin, melatonin, and nitric oxide. Such crosstalk events associated with OU-sensitive ATPases shall provide promising results comparable with those reported for Na^+^, K^+^-ATPase in animals. The possibility of the existence of sodium efflux systems in plant cells, with ouabain-binding sites and sequence homology to the Na^+^, K^+^-ATPase of animal cells, cannot be ruled out. In this context, the efflux tracer ouabain (sodium efflux inhibitor) and its fluorescent derivative 9-anthroylouabain can be employed to obtain successful physiological responses. A proteomic approach is also necessary for the identification of ouabain-binding proteins in plants. This shall clarify whether ouabain has a direct effect on some sodium efflux systems or acts through a secondary signaling via some regulatory proteins. The possibility of the involvement of ouabain in regulating the SOS components (Na^+^/H^+^ antiporters) shall provide new paradigms on the evolutionary significance of the ouabain-binding sites among sodium exchange systems in plants. The fact that P-type ATPases evolved differently among various groups of plants can be substantiated with evidence of sequence homology among ouabain-binding sites in sodium efflux proteins.

## Figures and Tables

**Table 1:** Role of various types of ion channels and transporters involved in sodium transport in plants.

Name	Role	Species	References
VI-NSCC (voltage-insensitive NSCC)	Na^+^-permeable conductance and influx into roots	*Arabidopsis thaliana*, *Secale cereal*, *Haemanthus*, *Clivia*, *Pisum sativum*, *Hordeum vulgare*, *Lotus japonicus*, *Triticum aestivum*	[44,45,46,50,51,52,53,54]
CNGC (cyclic nucleotide gated channel)	Unidirectional Na^+^ flux and Na^+^ uptake into roots	*Arabidopsis thaliana*, *Oryza sativa*	[55,58,59]
GLR (glutamate receptor)	Na^+^-permeable conductance and Na^+^ uptake into roots	*Arabidopsis thaliana*	[60,61,62,63]
HKT	Na^+^ influx in roots and Na^+^ retrieval from xylem	*Arabidopsis thaliana*, *Oryza sativa*, *Triticum turgidum*	[40,56,64,65,66,67,68,69]
HAK5	Na^+^-permeable conductance and low-affinity Na^+^ influx in roots	*Arabidopsis thaliana*, *Phragmites australis*	[56,70]
NHX	Na^+^ sequestration into vacuoles	*Brassica napus*, *Gossypium hirsutum*, *Zea mays*, *Oryza sativa*, *Nicotiana tabacum, Triticum aestivum*, *Arabidopsis thaliana*, *Cynodon dactylon*	[35,71,72,73,74,75,76,77,78]
SOS1	Na^+^ efflux from roots	*Cynodon dactylon*, *Arabidopsis thaliana*, *Suaeda salsa*	[35,79,80,81]
Ouabain-sensitive Na^+^, K^+^-ATPase	Na^+^ efflux from roots	*Daucus carota*, *Zea mays*, *Hordeum vulgare*, *Helianthus annuus*	[82,83,84,85]

## Abbreviations

NSCCs	Nonselective cation channels
NHX	Na^+^/H^+^ antiporter
SOS	Salt overly sensitive
OU	Ouabain
HAK	High-affinity potassium transporter
HKTs	High-affinity potassium transporter
CNGC	Cyclic nucleotide-gated channel
GLR	Glutamate receptor
VI-NSCC	Voltage-insensitive NSCC
PLC	Phospholipase C
CBL	Calcineurin B-like protein
CDPK	Calcium-dependent protein kinase
CIPK	CBL-interacting protein kinase
HMA	2-Hydroxymyristic acid
SnRK3	Sucrose non-fermenting-1 (SNF1)-related protein kinase 3
SCABP8	SOS3-like calcium binding protein 8
GI	Gigantea
ATP	Adenosine triphosphate
CLSM	Confocal laser scanning microscopy
EGTA	Ethylene glycol-*o*-*bis*(2-aminoethyl)*N*-tetraacetic acid
